# Molecular modeling of 7-propanamide benzoxaboroles as CPSF3 inhibitors through docking-based 3D-QSAR and molecular dynamics simulations

**DOI:** 10.1039/d5ra10104b

**Published:** 2026-07-02

**Authors:** Liyang Ji, Yiwei Liu, Guofeng Xu

**Affiliations:** a School of Pharmaceutical Sciences, Shanghai Jiao Tong University Shanghai 200240 China lyji2020@sjtu.edu.cn; b School of Chemical and Environmental Engineering and Shanghai Engineering Research Center of Green Fluoropharmaceutical Technology, Shanghai Institute of Technology Shanghai 201418 China yiweiliu@sit.edu.cn; c State Key Laboratory of Natural and Biomimetic Drugs, School of Pharmaceutical Sciences, Peking University Beijing 100083 China xuguofeng951006@outlook.com

## Abstract

Benzoxaboroles are boron-based heterocycles with broad therapeutic potential, including two FDA-approved drugs, tavaborole and crisaborole. It was reported that the discovery of 7-propanamide benzoxaboroles as novel potent anti-cancer agents, with compound 44 inhibiting cleavage and polyadenylation specific factor 3 (CPSF3), a key mRNA processing enzyme. By blocking CPSF3's RNA-binding function, compound 44 disrupts pre-mRNA cleavage and induces cancer cell death. In our current study, we systematically investigated the structure–activity relationships (SARs) of a series of 7-propanamide benzoxaboroles using docking-based three-dimensional quantitative structure–activity relationship (3D-QSAR). Comparative molecular field analysis (CoMFA, *r*^2^ = 0.937, *q*^2^ = 0.826) and comparative molecular similarity indices analysis (CoMSIA, *r*^2^ = 0.964, *q*^2^ = 0.882) reveals critical structural determinants for CPSF3 binding and inhibition. Additionally, molecular dynamics (MD) simulations were used to better understand the important molecular interactions between the inhibitors and CPSF3, such as metal-mediated coordination, Phe238-mediated pi–pi stacking, van der Waals interactions and hydrophobic embedding mediated by hydrophobic amino acids. Guided by the obtained SAR, we designed a series of seven novel derivatives featuring the 7-propanamide benzoxaborole scaffold. Among them, compounds 50 and 51 show enhanced pIC_50_ values compared to compound 32. In conclusion, this study explores SARs of benzoxaborole compounds through docking-based 3D-QSAR and MD simulations, offering valuable theoretical guidance for the rational design of new benzoxaborole derivatives as anti-cancer agents *via* inhibiting CPSF3.

## Abstract 2

This study explored SARs of benzoxaborole compounds through docking-based 3D-QSAR and MD simulations, offering valuable theoretical guidance for the rational design of new benzoxaborole derivatives as anti-MDA-MB-231 agents *via* inhibiting CPSF3.

## Graphical abstract

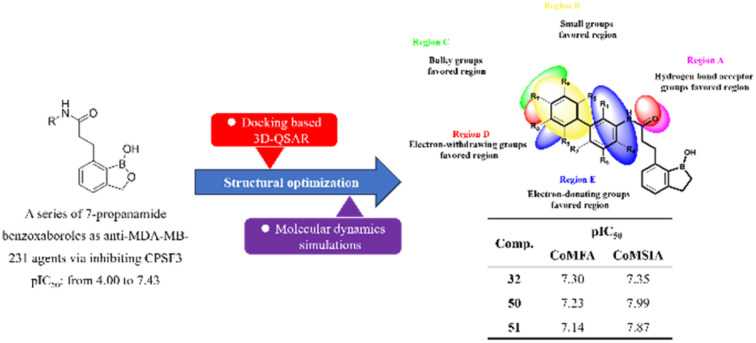

## 1 Introduction

**Fig. 1:**
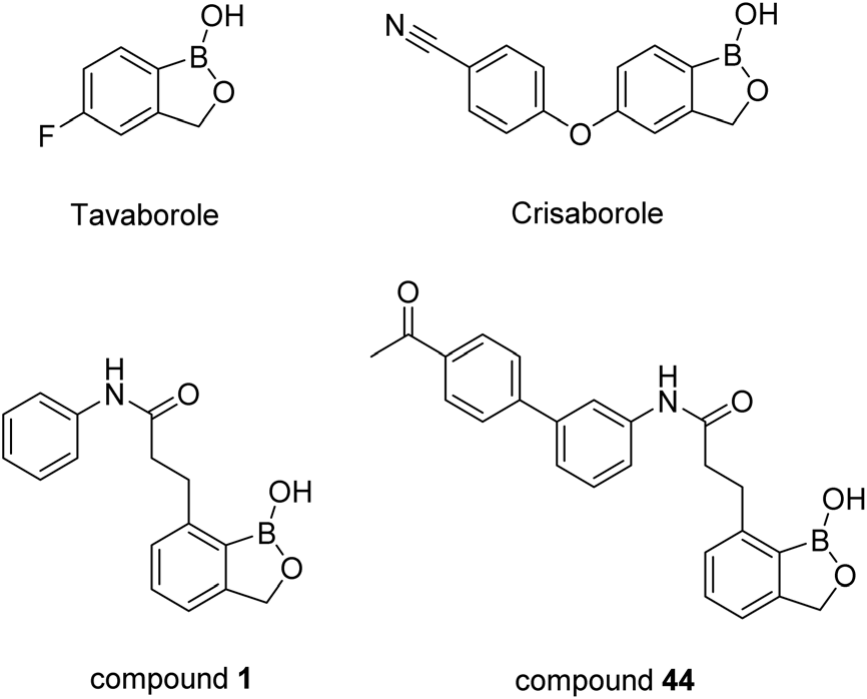
Structures of tavaborole (Kerydin), crisaborole (Eucrisa), 7-propanamide benzoxaboroles (compound 1 and compound 44).

Benzoxaboroles represent a class of boron-containing heterocyclic compounds distinguished by their unique chemical structures and biological activities. These compounds have garnered significant attention in medicinal chemistry due to their broad therapeutic applications, particularly in anti-cancer, anti-inflammatory, and anti-infective therapies.^1,2^ To date, two benzoxaborole-based drugs have been commercialized: tavaborole (Kerydin®), approved in 2014 for the topical treatment of onychomycosis,^3^ and crisaborole (Eucrisa®), approved in 2016 for the treatment of mild-to-moderate atopic dermatitis (Fig. 1).^4^

Our research, which centers on the anti-infective and anti-cancer applications of benzoxaborole-containing compounds led us in 2019 to report the identification of 7-propanamide benzoxaboroles as potent anti-cancer compounds.^5–9^ Through structural optimization of lead compound 1, we developed compound 44, the most potent analog in this series (Fig. 1). This compound displayed pronounced anti-cancer activity in both *in vitro* cell viability assays and *in vivo* cancer growth inhibition studies. Recently, it has been discovered that compound 44 exerted anti-cancer activity by specifically inhibiting CPSF3, disrupting its RNA-binding function and blocking mRNA processing, ultimately leading to cancer cell apoptosis.^10^ The target identification of compound 44 lays the foundation for the design and optimization of 7-propanamide benzoxaboroles as anti-cancer agents *via* inhibiting CPSF3.

CPSF3 is a crucial catalytic part of the cleavage and polyadenylation specificity factor complex.^11^ Its main role is to identify the cleavage site of pre-mRNA and facilitate the 3′-end cleavage, ensuring proper processing and maturation of mRNA.^12,13^ As an important regulator of gene expression, CPSF3 dysfunction or abnormal expression is linked to disease development, especially in cancers, where its overexpression has been shown to promote oncogenic traits such as cancer cell growth, survival, and spread.^14–16^ Certain cancers exhibit CPSF3-dependent pre-mRNA processing and transcriptional termination, including ovarian cancer, acute myeloid leukemia (AML), and Ewing's sarcoma.^17,18^ Because of CPSF3's vital role in mRNA processing, blocking its activity can interfere with gene expression in cancer cells, thereby inhibiting cancer growth. Considering the essential roles of CPSF in cancer pathogenesis, it has emerged as a promising therapeutic target for anti-cancer drug development.

In computer-aided drug design (CADD), three-dimensional quantitative structure–activity relationship (3D-QSAR) has become a pivotal computational approach, which establishes mathematical correlations between biological activities and molecular descriptors to guide rational drug optimization.^19–22^ Among the most widely employed 3D-QSAR techniques are comparative molecular field analysis (CoMFA) and comparative molecular similarity indices analysis (CoMSIA). In this study, utilizing our reported benzoxaborole derivatives, we developed CoMFA and CoMSIA models to elucidate their structure–activity relationships (SARs) against MDA-MB-231 cell line. Molecular dynamics (MD) simulations were also used to better understand the molecular interactions between the inhibitors and CPSF3. This study provides a valuable theoretical foundation for guiding structural optimization of benzoxaborole-containing CPSF3 inhibitors as anti-cancer agents.

## 2 Results and discussion

### 2.1. Bioactive conformation modeling

#### 2.1.1. Acquisition of bioactive conformations

The molecular docking was performed to obtain bioactive conformations for subsequent 3D-QSAR modeling. We first conducted structural refinement of all small molecules under physiological conditions to more accurately simulate their binding behaviour with the target site. The reason is that boron in the benzoxaborole scaffold bound to the target's active site in a tetrahedral anionic form, according to the crystal structure study of the CPSF3-compound 44 complex.^10^ Then, all compounds were docking into the CPSF3 binding site. We allowed ten docking conformations to be output per compound. The selection criterion for bioactive conformation requires maintenance of conformational and spatial orientation consistency between the output conformations of each compound and the crystallographic conformation of compound 44, as determined by Glide docking scores and analysis of protein-ligand interactions.

#### 2.1.2. Molecular alignment

**Fig. 2:**
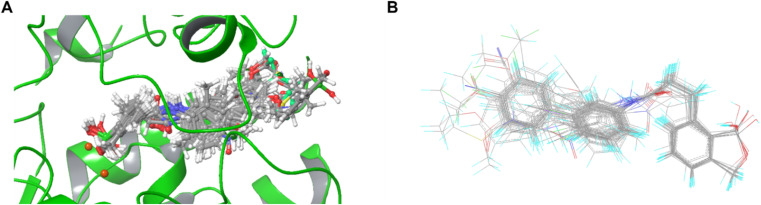
Molecular alignment of all 44 compounds based on their docking conformations. (A) Alignment within the binding pocket (PDB ID: 8T1Q); (B) alignment results in SYBYL-X.

For a same compound series, biological activity data are strongly linked to various substitutions at a specific point. Therefore, the quality of 3D-QSAR models depends on molecular alignment. Although the common structure is usually used for molecular alignment, it is probably more valid to construct and evaluate the 3D-QSAR models using the active conformations.^23^ This reflects the real situation where different bioactive conformations are adopted by these derivatives because of various substituents. Furthermore, alignment using docking conformations also helps in understanding the contour maps of the models in a structure-based manner. The molecular alignment of all compounds based on their docking-based bioactive conformations is shown in Fig. 2.

While docking-based alignment is prioritized for its biological relevance, the sensitivity of 3D-QSAR to different alignment protocols remains an inherent characteristic of the methodology. To further validate our choice, we performed a brief internal comparison, which showed that the docking-based alignment yielded a more consistent field distribution corresponding to the known structural pharmacology of CPSF3 compared to a rigid scaffold fit.

#### 2.1.3. 3D-QSAR statistics

In our previous studies, this class of compounds demonstrates similar SAR trends in their anti-proliferative effects against MDA-MB-231 cells compared to SKOV3 and HCT-116 cells.^9^ Key structural features, including the benxozaborole scaffold, the 7-propanamide flexible side chain (particularly its position), and the central benzene ring, were found to be crucial for anti-cancer activity.^9,24^ These structural elements have been experimentally validated to contribute significantly to the compounds' inhibitory effects on SKOV3 and HCT-116 cell proliferation through binding to CPSF3 protein.^10,17^ Therefore, we propose that all 44 compounds developed from the lead compound 1 (which incorporates these critical structural characteristics) in the current study are highly likely to exert anti-proliferative effects on MDA-MB-231 cells *via* the same mechanism of CPSF3 binding and inhibition. The 3D-QSAR (including CoMFA and CoMSIA methods) modeling of 7-propanamide benzoxaboroles as anti-MDA-MB-231 agents were constructed by SYBYL-X, as shown in Table S1. The statistical performance of the 3D-QSAR models is presented in Table 1.

**Table 1:** Internal and external validation results of CoMFA and CoMSIA models

Statistical parameters	CoMFA	CoMSIA
**Original PLS**		
*q* ^2^ a	0.826	0.882
*N* b	3	8
*r* ^2^ c	0.937	0.989
SEE^d^	0.240	0.107
*F* e	147.969	293.634

**Scrambling test**		
c*Q*^2^^f^	0.635	0.585
CSDEP^g^	0.571	0.661
d*q*^2^/d*r*^2^*yy*^h^	1.560	2.211
*N* i	500	500

**External validation**		
*r*_pred_^2^ j	0.941	0.995
*r*_m_^2^_(test)_ k	0.755	0.816
Δ*r*_m_^2^^l^	0.052	0.044
SDEP_ext_^m^	0.412	0.314

**Fraction of field contributions**		
Steric	0.636	0.195
Electrostatic	0.364	0.224
Hydrogen bond donor	—	0.243
Hydrogen bond acceptor	—	0.338

a Cross-validation correlation coefficient (*q*^2^).

b The optimal number of components (*N*).

c Coefficient of determination (*r*^2^).

d Standard error of estimate (SEE).

e *F*-test value (*F*).

f Corrected *Q*^2^ (c*Q*2).

g Cross-validated standard deviation of error of prediction (CSDEP).

h The derivative of *q*^2^ with respect to *r*^2^ (d*q*^2^/d*r*^2^*yy*).

i Number of iterations (*N*).

j External validation determination coefficient (*r*_pred_^2^).

k Modified *r*^2^ (*r*_m (test)_^2^).

l Average *r*_m_^2^ difference (Δ*r*_m_^2^).

m External standard deviation error of prediction (SDEP_ext_).

Specifically, the CoMFA model shows a cross-validated coefficient (*q*^2^) of 0.826 using three optimal components, alongside a non-cross-validated correlation coefficient (*r*^2^) of 0.937. The model's reliability is supported by a standard error of estimate (SEE) of 0.240 and an *F*-value of 147.969. Steric descriptors contributed 63.6% to the total variance, while electrostatic descriptors account for 36.4%, identifying steric interactions as the primary influence on the SAR.

The CoMSIA model was constructed by considering five molecular fields: steric (S), electrostatic (E), hydrophobic (H), hydrogen bond donor (D), and hydrogen bond acceptor (A). Among the various combinations, the CoMSIA-SEDA model was identified as the best model. This model, employed for our subsequent detailed analysis, shows a q^2^ of 0.882 using eight optimal components, alongside *r*^2^ of 0.989. The model's high statistical reliability is further supported by a SEE of 0.107 and an *F*-value of 293.634. The corresponding field contributions are 19.5%, 22.4%, 24.3%, and 33.8% for steric, electrostatic, hydrogen bond donor, and acceptor fields, respectively. It can be found that the hydrogen bond acceptor field makes great contributions to the protein–ligand binding and anti-MDA-MB-231 activity, especially.

To evaluate the risk of chance correlation and the robustness of the 3D-QSAR studies, Y-scrambling test (also named scrambling stability test in SYBYL-X) was systematically performed for both models. Initially, the CoMFA model was subjected to this validation, yielding a high sensitivity slope (d*Q*^2^/d*R*^2^) of 1.560, a CSDEP of 0.571, and a c*Q*^2^ of 0.635. Subsequently, a parallel analysis was conducted for the CoMSIA model to ensure its correlation is not due to chance; this yielded an even higher sensitivity slope (d*Q*^2^/d*R*^2^) of 2.211, a CSDEP of 0.661, and a c*Q*^2^ of 0.585. Also, the low intercepts of c*Q*^2^ obtained from the scrambling test indicate that the model's predictive power is derived from genuine SAR rather than chance correlation (see Fig. S1). Collectively, these parameters demonstrate that both the CoMFA and CoMSIA models are statistically robust and possess genuine predictive power, rather than being the result of fortuitous data alignment.

#### 2.1.4. Validation of the 3D-QSAR models

To assess the external predictive power of the models, a test set of 10 compounds was used. Both the CoMFA and CoMSIA models were employed to calculate their predicted activity values. As shown in Table S1, the predicted pIC_50_ values for the majority of the compounds show excellent agreement with the experimental data within a reasonable error range, except for compounds 1 and 2. The residual values between the experimental pIC_50_ and predicted pIC_50_ by the 3D-QSAR models for both compounds exceeded 0.6 log units. We propose that the primary source of error stems from a misalignment between the actual mechanism of action and the model's assumptions. As cellular activity data were employed, CPSF3 inhibition cannot be definitively established as the exclusive target for all compounds' anti-proliferative effects in MDA-MB-231 cells. Compounds 1 and 2, in particular, may engage additional critical targets within this cell line.

Also shown in Table 1, the CoMFA model gave a predictive correlation coefficient *r*_pred_^2^ of 0.941, with a modified term *r*_m (test)_^2^ of 0.755, Δ*r*_m_^2^ of 0.052, and SDEP_ext_ value of 0.412.^25,26^ The CoMSIA model gave a predictive correlation coefficient *r*_pred_^2^ of 0.995, with a modified term *r*_m (test)_^2^ of 0.816, Δ*r*_m_^2^ of 0.044, and SDEP_ext_ value of 0.314. Comparatively, the CoMSIA model shows better capability in predicting the activity of external compounds.

**Fig. 3:**
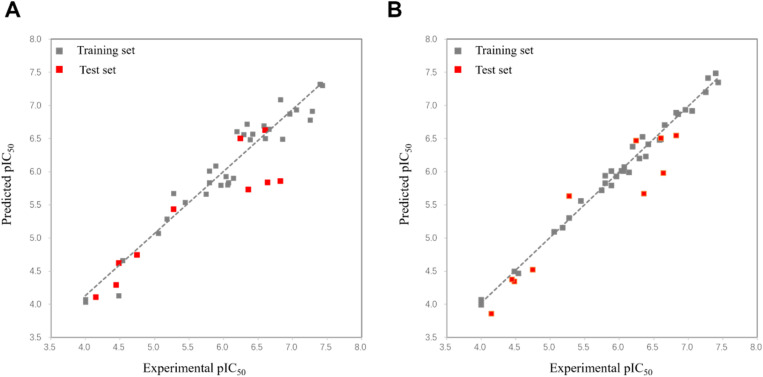
Correlation between experimental and predicted pIC_50_ values for the (A) CoMFA and (B) CoMSIA models.

The correlation between experimental and predicted values for the benzoxaborole derivatives in this study was calculated. As shown in Fig. 3, the data points for both the training and test sets are closely distributed along the line *Y* = *X*, demonstrating a strong correlation between the two values. This alignment supports the high predictive accuracy and robustness of both the CoMFA and CoMSIA models.

#### 2.1.5. Contour maps analysis

**Fig. 4:**
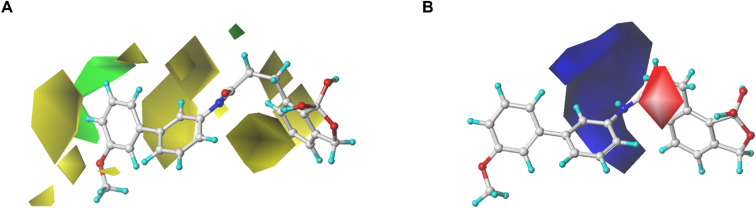
CoMFA 3D-contour maps showing the (A) steric and (B) electrostatic fields, with compound 32 used as a structural template. Green regions indicate where sterically favored regions for enhancing anti-MDA-MB231 activity, while yellow regions indicate sterically unfavored regions are. The blue region indicates where electron-donating groups are beneficial for enhancing activity, and the red region indicates where electron-withdrawing groups are favored.

**Fig. 5:**
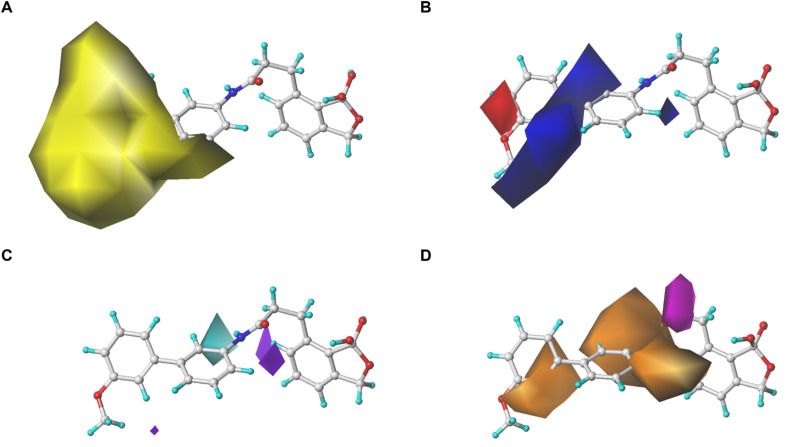
CoMSIA 3D-contour maps showing the (A) steric, (B) electrostatic, (C) hydrogen bond donor, and (D) hydrogen bond acceptor fields, with compound 32 as the structural template. The yellow region indicates a sterically unfavored region. The blue region favors electron-donating groups, while the red regions favor electron-withdrawing groups. The cyan region favors hydrogen bond donor groups, while the purple region indicates an unfavored region. Magenta regions favor hydrogen bond acceptor groups, while orange regions indicate where hydrogen bond acceptor groups are unfavored.

To visualize the SAR of the benzoxaborole derivatives, 3D contour maps were generated from the QSAR models. As illustrated in Fig. 4 and 5, compound 32, which shows the most potent anti-MDA-MB-231 activity, was used as a structural template to map the features of fields defined by the CoMFA and CoMSIA models.

The contour map of the steric field of CoMFA is shown in Fig. 4A, with the effect of the steric field on activity indicated by green and yellow colors. Green regions around the compound indicate that groups with larger connected spaces may enhance the compound's activity, while yellow regions indicate that such groups might decrease activity. From Fig. 4A, we can see that the green region covers the *meta*- and *para*-positions on the terminal benzene ring of the compound, indicating that introducing a slightly bigger group in both positions would be beneficial for enhancing the compound's activity. The contour map of the electrostatic field of CoMFA is shown in Fig. 4B, indicating the effect of the electrostatic field on activity with blue and red colors. The blue region covers the central benzene ring of the compound, indicating that introducing an electron-donating group in this position might enhance the compound's activity. The red region indicates that the carbonyl moiety in the 7-propionamide side chain functions as an electron-withdrawing group, promoting enhancement of activity.

The contour map of the steric field of CoMSIA is shown in Fig. 5A. Unlike the CoMFA model predictions, introducing a bulky group at the central benzene ring of 7-propanamide benzoxaboroles may lead to reduced activity. Owing to the distinct mathematical frameworks underpinning CoMFA and CoMSIA, the observed divergence in their contour maps should be interpreted as a complementary alignment of their respective strengths. CoMFA, employing the steeply defined Lennard-Jones potential, excels at identifying precise steric constraints for localized functional group optimizations. Conversely, the Gaussian-based distance dependence of CoMSIA facilitates a smoother capture of global property distributions, thereby providing a more holistic perspective on large-scale SAR trends. The contour map of the electrostatic field of CoMSIA is shown in Fig. 5B. The red region around the *para*-position of the terminal benzene ring indicates that introducing an electron-withdrawing group at this position may enhance the compound's activity. The blue regions near the *ortho*-position of both the central and terminal benzene ring indicate that introducing an electron-donating group at both positions may enhance activity, similar to the pattern seen in the CoMFA model. The contour map of the hydrogen bond donor field of CoMSIA is shown in Fig. 5C. The cyan region indicates where introducing a hydrogen bond donor group may enhance the compound's activity, while the purple regions indicate where introducing a hydrogen bond donor group may reduce the compound's activity. This result indicates that the hydrogen atom binds to the nitrogen in the amide bond can serve as a hydrogen bond donor, thereby enhancing the interaction of these inhibitors with CPSF3. The contour map of the hydrogen bond acceptor field of CoMSIA is shown in Fig. 5D. The magenta region indicates where introducing a hydrogen bond acceptor group may enhance the compound's activity, while orange regions indicate where hydrogen bond acceptor groups were unfavored. From Fig. 5D, we can see that the presence of the magenta region enveloping the carbonyl group of the compound 32, indicating that the carbonyl moiety in the 7-propionamide side chain functions as a hydrogen-bond acceptor, playing a critical role in mediating anti-proliferative activity against MDA-MB-231 cells.

#### 2.1.6. SAR summary

**Fig. 6:**
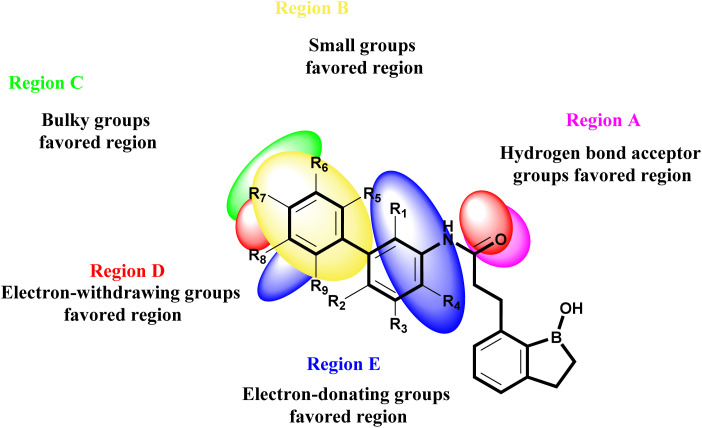
SAR diagram of 7-propanamide benzoxaboroles as anti-MDA-MB231 agents.

Analysis of the CoMFA and CoMSIA results established the SAR profile for 7-propanamide benzoxaboroles as inhibitors of the MDA-MB-231 cell line. As shown in Fig. 6, key findings indicate that inhibitory activity is enhanced by the introduction of specific substituents within distinct molecular regions: a hydrogen bond acceptor moiety (*e.g.*, carbonyl group^27^) in Region A, a small substituent (*e.g.*, methyl group^28^) in Region B, a moderately bulky group (*e.g.*, methoxy group^29^) in Region C, an electron-withdrawing group (*e.g.*, trifluoromethyl group^30^) in Region D and an electron-donating group (*e.g.*, methoxy group^31^) in Region E.

### 2.2. Molecular dynamics simulations

Compared to the lead compound 1, compound 44 has a phenyl substituent in the small groups favored region and an acetyl substituent in the electron-withdrawing groups favored region (R_7_), and there is significantly increased anti-cancer potency. To investigate the effect on stability and activity made by substituents in these two regions, compounds 1 and 44 were selected to conduct the molecular docking and dynamics simulation with CPSF3. MD trajectory analysis and binding free energy calculation of both compounds provide detailed information about the differences between them.

#### 2.2.1. Comparison of docking results for compounds 1 and 44

Molecular docking is a pivotal tool in molecular modeling for evaluating the energetic interactions and orientations of ligands within a receptor's active site. However, as a static calculation, docking provides limited insights into the temporal behavior of the system.^32^ To address this, molecular dynamics (MD) simulations are frequently employed alongside docking to probe the molecular flexibility of both ligands and receptors. Given that certain conformational transitions occur on nanosecond timescales, the direct application of MD to extensive virtual screening may be computationally prohibitive. Therefore, a strategic approach—selecting promising configurations from docking studies for subsequent MD analysis—is crucial to enhance the theoretical accuracy and viability of the drug design workflow. A successful research case is the study of histone deacetylase 1 (HDAC1) inhibitors.^33^ Through molecular docking and MD analysis of HDAC1 enzyme with ligands and the co-crystal inhibitor (PDB: 1C3S), this study revealed key differences and similarities in binding interactions.

**Fig. 7:**
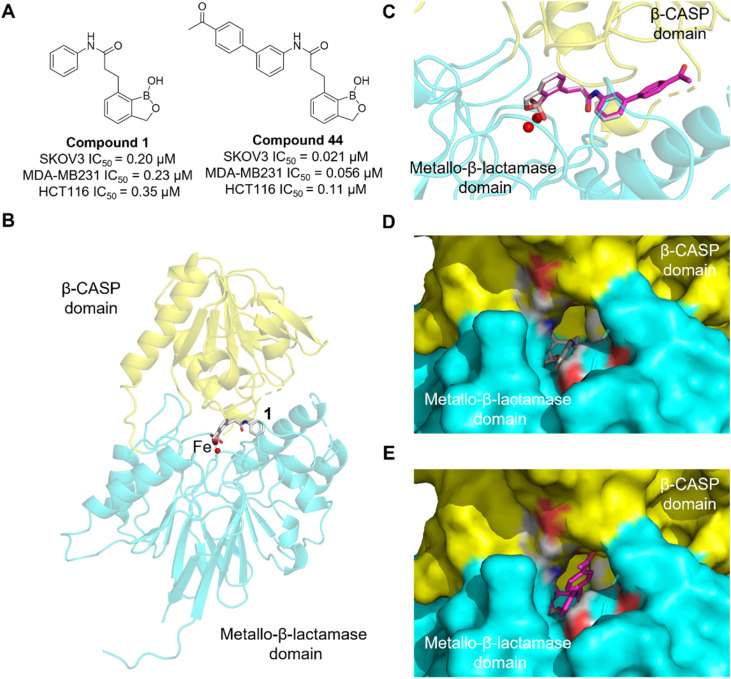
Docking results for compound 1 and compound 44 against CPSF3 (PDB ID: 8T1Q). (A) Chemical structures of compound 1 and compound 44. (B) Global architecture of the human CPSF3-compound 1 complex. The metallo-β-lactamase and β-CASP domains of CPSF3 are highlighted in cyan and yellow, respectively. Compound 1 is rendered as a stick model (carbon in gray, boron in pink), while the dual catalytic iron (Fe^2+^) ions within the active site are depicted as red spheres. (C) Docking poses of compound 1 and compound 44 against CPSF3 based on the alignment. Compound 1 and compound 44 are shown as stick models, with carbon atoms in gray and magenta, respectively. (D) Electrostatic surface of the CPSF3-compound 1 complex, highlighting an opening that permits access for the compound to the cavity in the active site region. (E) Electrostatic surface of the CPSF3-compound 44 complex, revealing a similar opening compared to that of the CPSF3-compound 1 complex.

Similarly, to investigate the effect on anti-cancer activity made by substituents in these two regions, we first examined the docking structures of both compounds against CPSF3 (Fig. 7). As shown in Fig. 7A, structural optimization of compound 1 yielded compound 44, resulting in a significant increase in anti-cancer activity. Similar to the co-crystal inhibitor (PDB ID: 8T1Q), compound 1 shows clearly defined electron density and binds within the active site of CPSF3, positioned at the interface of its metallo-β-lactamase and β-CASP domains, as shown in Fig. 7B. The docking pose of compound 44 closely resembles that of compound 1, as shown in Fig. 7C. This consistency arises because the two Fe ions in the active site forms metal-coordination bonds with both the protein residues and ligands, serving as an interoperable anchor that stabilizes the position of 7-propanamide benzoxaboroles. The introduction of a 4-acetylphenyl group significantly enhances bioactivity, indicating that the positive contribution of the 4-acetyl moiety, functioning as both an electron-withdrawing group and a slightly bulky group, substantially outweighs the negative impact arising from the overall steric bulk of the 4-acetylphenyl group (Fig. 6). CPSF3 adopts a sequestered, inactive conformation that effectively shields the bound inhibitor from the surrounding solvent.^10^ However, as shown in Fig. 7D, a surface-accessible channel remains, providing the inhibitor with entry into an expansive cavity between the interdomain interface. Notably, the 4-acetylphenyl tail of compound 44 is oriented toward the rim of the active site, as shown in Fig. 7E, indicating that this tail could interact with additional residues, thereby accounting for the exceptional inhibitory activity exhibited by compound 44. Prime MM-GBSA calculations in Schrödinger were also performed, revealing that the binding free energy of compound 44 is −79.98 kcal mol^−1^, while that of compound 1 is −59.27 kcal mol^−1^, indicating that compound 44 binds more tightly to CPSF3 compared to compound 1, which correlates with their inhibitory activity.

#### 2.2.2. Comparison of MD trajectory results for compounds 1 and 44

**Fig. 8:**
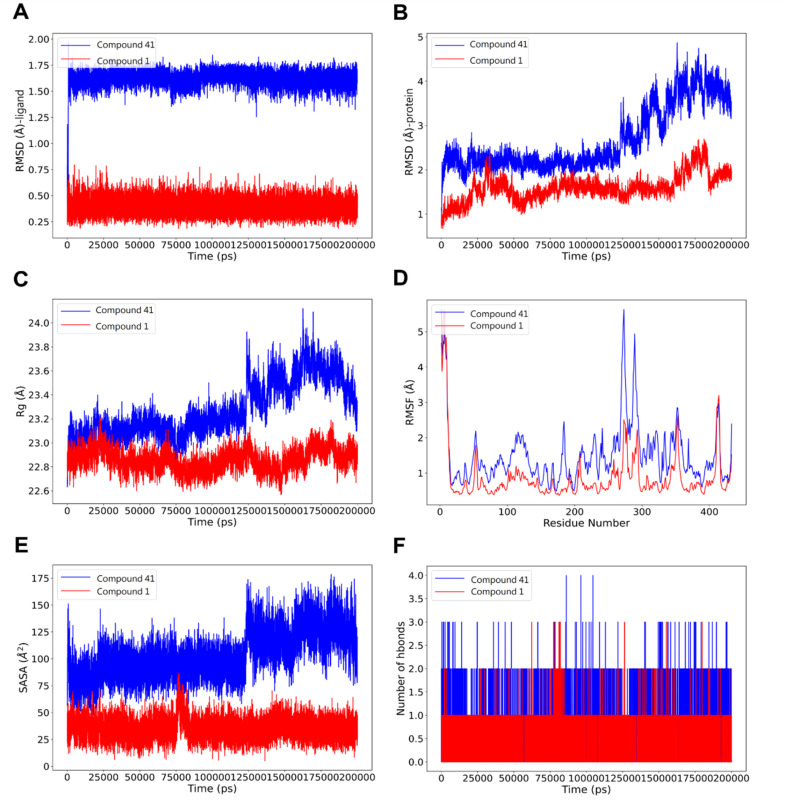
RMSD-ligand (A), RMSD-protein (B), *R*_g_ (C), RMSF (D), SASA (E), and Number of H-bonds (F) for CPSF3-compound 1 and CPSF3-compound 44 complexes during the 100 ns MD simulation. The CPSF3-compound 1 complex and CPSF3-compound 44 complex are shown in red and blue, respectively.

Traditional methods (*e.g.*, molecular docking) lack temporal resolution, whereas integrating MD simulations enables more comprehensive analysis of dynamic interactions. To explore the dynamic stability of both systems, 200-ns molecular dynamics (MD) simulations using the docking structure of compounds 1 and 44 were performed. The resulting trajectories are shown in Fig. 8. As shown in Fig. 8A and B, the root-mean-square deviation (RMSD) plots of both complexes show some fluctuation, but the overall actual values are small, indicating that both the CPSF3-compound 1 and CPSF3-compound 44 complexes stay in a relatively stable and integrated state. Compound 44 shows larger fluctuations throughout the simulation compared to compound 1, indicating reduced stability. This phenomenon is likely attributable to the introduction of a biphenyl moiety and 4-acetyl modification, which collectively enhance ligand flexibility. Excessive flexibility might incur a substantial conformational entropy penalty upon binding to the target protein, thereby weakening the apparent binding affinity (Δ*G*_bind_).^34^ This interpretation aligns with the SAR presented in Fig. 6, wherein the biphenyl incorporation fails to contribute positively to activity enhancement. Furthermore, the increased ligand flexibility correlates with increased radius of gyration (*R*_g_, Fig. 8C), root mean square fluctuation (RMSF, Fig. 8D) and solvent accessible surface area (SASA, Fig. 8E) values in the CPSF3-compound 44 complex relative to the CPSF3-compound 1 complex, reflecting mutual conformational adaptation during protein–ligand recognition. Notably, the range of residues 250–300 of CPSF3 in CPSF3-compound 44 complex shows a peak RMSF value exceeding 5 Å, indicating that compound 44 binding might induce allosteric dynamics in distal secondary structures such as β-sheets. Additional simulation was run to guarantee the statistical accuracy of the MD results (see Fig. S2). The relative trends in RMSD are similar, while those in RMSF show a reversal; this is likely due to the MD simulations not yet having reached a state of equilibrium.

Generally, higher complex stability correlates with lower binding free energy and enhanced bioactivity. Compared to compound 1, compound 44 shows significantly enhanced activity with reduced stability. This apparent inverse correlation between simulated stability and biological potency had prompted our focused investigation. We proposed that the acetyl group in compound 44 plays a critical role in this phenomenon. Acting as a hydrogen-bond acceptor, it forms a key hydrogen bond interaction with amino acid residues in the active pocket. This interaction might stabilize the transition state during the compound 44 binding process, thereby enhancing the association rate and overcoming the ground-state instability.^35^ As shown in Fig. 8F, the CPSF3-compound 1 complex maintains only a single H-bond during the majority of the simulation time. In contrast, the CPSF3-compound 44 complex maintains two persistent H-bonds throughout most of the simulation, indicating significantly stronger hydrogen bonding than observed with compound 1, which supports the formation of specific hydrogen-bonding interactions mediated by the acetyl moiety.

It is noteworthy that compound 44 shows higher conformational flexibility during MD trajectories. While this might initially suggest reduced stability, such fluctuations often reflect the dynamic nature of the protein-ligand interface. We must exercise caution in over-interpreting these stability indicators, as the simplified force field parameters may not fully capture the complex electronic environment of the binding site. Therefore, the experimental bioactivity remains the primary criterion for assessing the efficacy of compound 44, while the MD trajectory provides a qualitative glimpse into its potential binding adaptability.

#### 2.2.3. Binding free energy calculation

Binding free energy (Δ*G*_bind_) is a key metric for compound activity, where lower values (typically in kcal mol^−1^) correlate with enhanced bioactivity. To evaluate the binding affinity of each complex, the MM/PBSA method was used to calculate the binding free energies. As shown in Table 2. We can see that the total binding free energies of compounds 1 and 44 are −91.407 kcal mol^−1^ and −104.989 kcal mol^−1^, respectively. Compound 44 shows a lower (more negative) total free energy of binding, indicating that it binds CPSF3 significantly better than compound 1, which is consistent with their biological activities. Notably, the electrostatic components (Δ*E*_ele_) were calculated at −316.631 kcal mol^−1^ and −324.036 kcal mol^−1^, respectively. These values are significantly more negative than the other energy terms, indicating that electrostatic interactions represent the dominant driving force in the ligand-binding process. The value of Δ*E*_vdw_ for compound 1 is +12.438 kcal mol^−1^ (weakly unfavorable), indicating that the van der Waals interaction is also weakly unfavorable. The value of Δ*E*_vdw_ for compound 44 is −0.072 kcal mol^−1^, which shifts the van der Waals interaction from unfavorable to weakly favorable compared to compound 1, indicating that the structural modification from compound 1 to compound 44 optimizes binding to CPSF3. The solvation free energies (Δ*G*_sol_) of the two systems are close, but the slight disadvantage of polar solvation (Δ*G*_epb_) is offset by the slightly better nonpolar solvation contribution (Δ*G*_enpolar_) of compound 44. The introduction of a 4-acetylphenyl group in compound 44 results in a significantly higher entropic penalty (−*T*Δ*S* = 16.179 kcal mol^−1^) compared to compound 1 (−*T*Δ*S* = 9.946 kcal mol^−1^), likely due to the loss of greater conformational freedom associated with the expanded biphenyl scaffold upon binding. Additional simulation was run to guarantee the statistical accuracy of the binding energy (Δ*G*_bind_), which shows the similar trends (see Table S2).

**Table 2:** Calculated binding energy (kcal mol^−1^) for compounds 1 and 44 binding to CPSF3^a^^,^^b^^,^^c^

Terms	CPSF3-compound 1	CPSF3-compound 44
Δ*E*_vdw_	12.438	−0.072
Δ*E*_ele_	−316.631	−324.036
Δ*G*_epb_	206.356	207.509
Δ*G*_enpolar_	−3.516	−4.570
Δ*E*_gas_	−304.193	−324.108
Δ*G*_sol_	202.840	202.939
−*T*Δ*S*	9.946	16.179
Δ*G*_bind_	−91.407	−104.989

a Δ*E*_gas_ = Δ*E*_ele_ + Δ*E*_vdw_.

b Δ*G*_sol_ = Δ*G*_epb_ + Δ*G*_enpolar_.

c Δ*G*_bind_ = Δ*E*_gas_ + Δ*G*_sol_ − *T*Δ*S*.

**Fig. 9:**
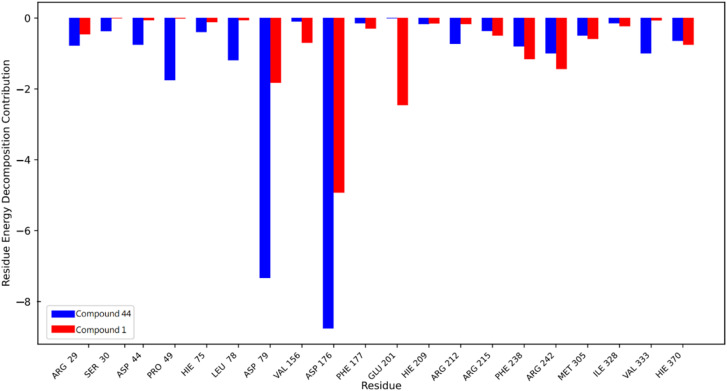
Binding free energy decomposition plots for the two systems.

**Fig. 10:**
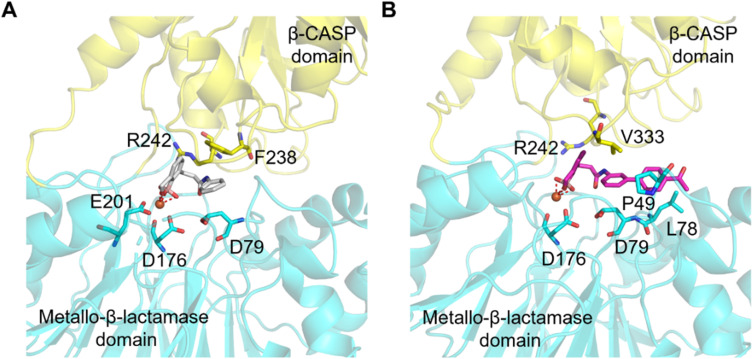
Average structures of CPSF3 with compounds 1 (A) and 44 (B). The metallo-β-lactamase and β-CASP domains of CPSF3 are shown in cyan and yellow, respectively. The bonds of residues and ligands are shown as sticks, and the carbon atoms of compounds 1 and 44 are shown in gray and magenta, respectively. The red dotted line indicates metal coordination.

To better understand the structural basis of the observed SAR, per-residue energy patterns were examined. As shown in Fig. 9, while compound 1 primarily binds to Asp79, Asp176, Glu201, Phe238, and Arg242, compound 44 additionally interacts with Pro49, Leu78, and Val333 while sharing the common residues Asp79, Asp176, and Arg242. To further observe the orientation of compounds and the position of these residues, we extracted the structure of the final state. As shown in Fig. 10, it is apparent that compounds 1 and 44 adapt similar binding poses, surrounded by these residues. The Fe ion within the active site functions as a central coordination hub, forming critical interactions with both the protein residues and the ligand to maintain the precise three-dimensional architecture of the binding pocket. This structural stabilization is essential for preserving the correct binding mode and interaction network during the MD simulation, which directly influences the accurate calculation of binding free energies. The favorable contributions are attributed to Asp79, Asp176, and Glu201, indicating that salt bridge networks play a supportive role in anchoring the ligand. Notably, the interaction profile of Phe238 offers a qualitative explanation for the importance of the terminal benzene ring in compound 1; the predicted stacking appears to be a critical factor that, when disrupted by electron-withdrawing substituents, aligns with the experimentally observed decrease in potency. Additionally, the cluster of Pro49, Leu78, and Val333 provides a hydrophobic-rich pocket that likely acts as a structural scaffold, promoting the optimal burial of the scaffold of compound 44 within the binding site. In addition, we found a larger cavity between the two structural domains of the CPSF3-compound 44 complex compared to the CPSF3-compound 1 complex, indicating that the benzoxaborole compound might bind to CPSF3 *via* an induced-fit mechanism, inhibiting its function by occupying the catalytic pocket and inducing local structural rearrangements (*e.g.*, active site closure or side chain displacement).

In summary, the binding mode of 7-propanamide benzoxaboroles within the CPSF3 active pocket shows an electrostatically driven mechanism complemented by extensive hydrophobic interactions. These findings indicate that structural optimization should prioritize introducing polar substituents to modulate global/local electron density, while incorporating hydrophobic groups complementary to the pocket to enhance van der Waals interaction and hydrophobic burial.

### 2.3. Design new CPSF3 inhibitors

Guided by the SAR from 3D-QSAR models, we designed a series of seven novel derivatives featuring the 7-propanamide benzoxaborole scaffold. These compounds, were developed by strategically introducing various substituents at different positions on the terminal benzene ring of compound 32, which may intend as potential CPSF3 inhibitors (see Table 3). Compounds 45–48 were designed by adding different electron-withdrawing groups in the R7 position to form favorable electrostatic interactions. Compounds 49–51 were designed by introducing an electron-withdrawing group in the R7 position and an electron-donating group in the R8 or R9 position to promote synergistic enhancement of activity. The pIC_50_ values of the designed compounds were predicted using the built CoMFA and CoMSIA models. As shown in Table 3, all the designed compounds show satisfactory inhibitory activity targeting CPSF3 compared to compound 32, and the predictive values are in accordance with the summarized SARs.

**Table 3:** Structures of newly designed 7-propanamide benzoxaboroles and their predicted pIC_50_ values against MDA-MB-231 cells

Comp.	Structure	Pred. (CoMFA)	Pred. (CoMSIA)
32	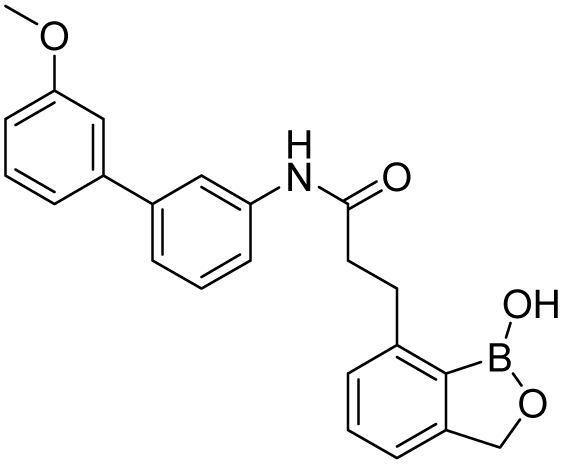	7.30	7.35
45	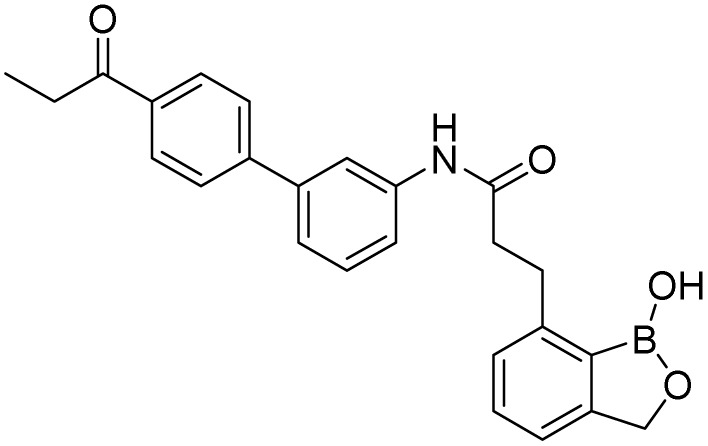	6.93	7.30
46	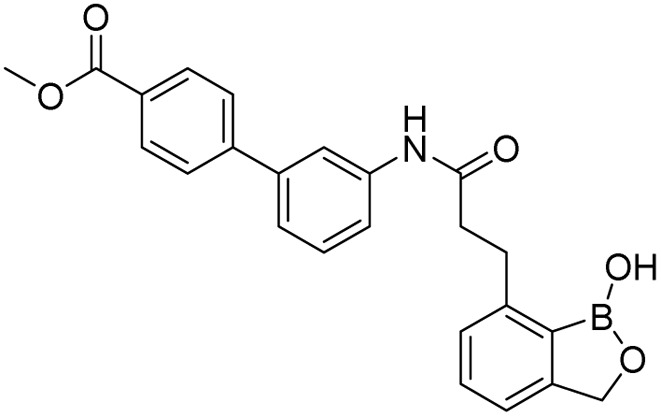	6.83	7.29
47	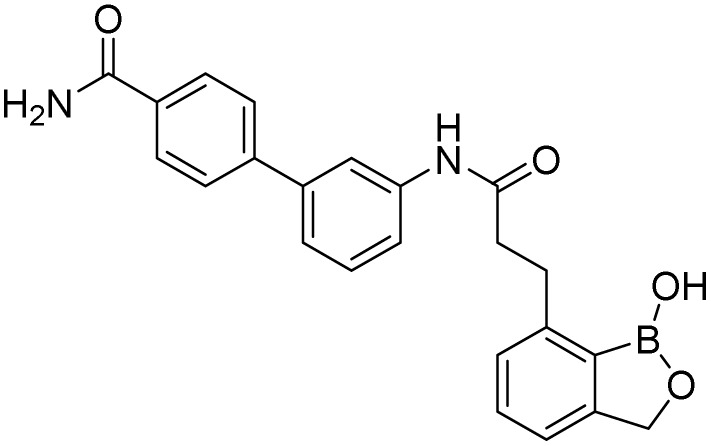	6.84	7.41
48	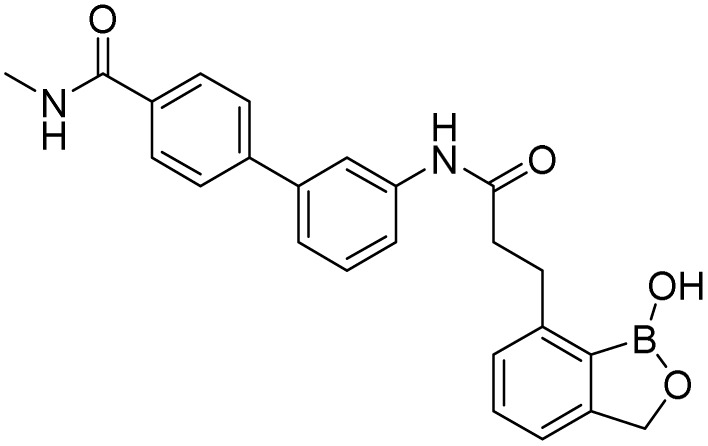	6.87	7.34
49	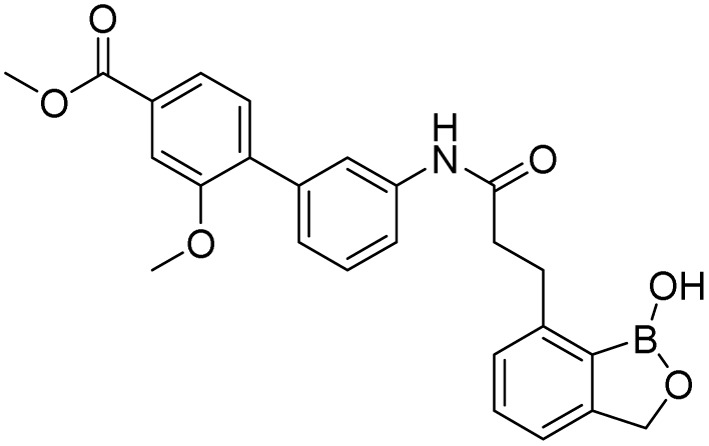	7.02	7.28
50	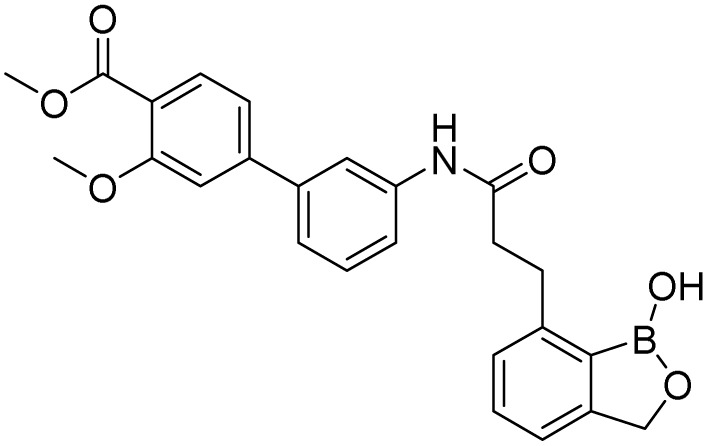	7.23	7.99
51	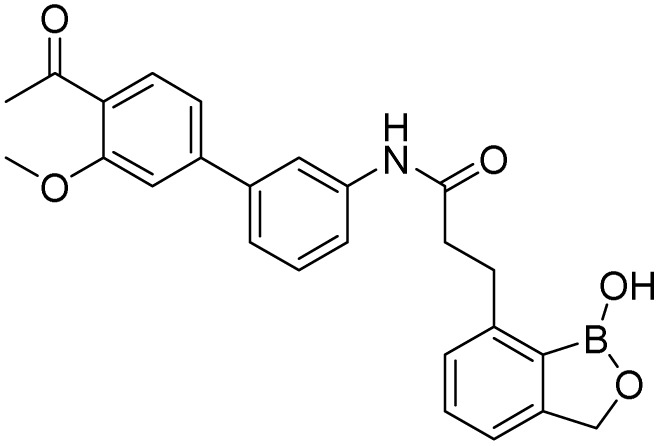	7.14	7.87

## 3 Experimental

### 3.1. 3D-QSAR study

#### 3.1.1. Data set for 3D-QSAR models

A dataset comprising 44 7-propanamide benzoxaboroles was used in this study, previously reported by our group.^9^ The inhibitory activities against MDA-MB-231 cells were determined under uniform experimental conditions, thus providing the benefit for data consistency. Biological activity data (IC_50_) for the MDA-MB-231 cell line (obtained from the American Type Culture Collection, ATCC) were taken from ref. 9. The cells were cultured in Dulbecco's modified Eagle's medium (DMEM) complete growth medium (high-glucose DMEM medium, supplemented with 10% fetal bovine serum, 100 mg per mL streptomycin, and 100 units per mL penicillin). For the 3D-QSAR analysis, IC_50_ values were transformed into their negative decadic logarithms pIC_50_ (pIC_50_ = −log_10_(IC_50_)) to serve as the dependent variable. This conversion enhances data linearity and model predictability. The resulting pIC_50_ values range from 4.00 to 7.43, providing a broad and well-distributed activity profile ideal for robust statistical modeling.

#### 3.1.2. Molecular docking

The co-crystal structure of CPSF3 with compound 44 (PDB ID: 8T1Q) was obtained from the Protein Data Bank (PDB) and used as the docking template. The crystal structure was preprocessed using the Protein Preparation Wizard within the Schrödinger Maestro suite (Schrödinger, LLC, New York, NY, 2024).^36^ This preparation involved the removal of crystallographic water molecules, as well as the addition of missing hydrogen atoms, the assignment of protonation states and partial charges. The OPLS_2005 force field^37^ was used to conduct above structural refinements. A grid box centered on the native ligand and matched in size was then generated to define the CPSF3 binding pocket. 3D-structures of all compounds were prepared with the LigPrep module^38^ from Schrödinger, and their ionization states were determined with Epik^39^ at pH 7.4 ± 2.0. Finally, the compounds were docked into the CPSF3 binding pocket and evaluated using Glide's extra precision (XP) mode.^40^ The Schrödinger docking score^41^ served as the scoring function. All other parameters were set to default. The most likely bioactive conformation for each compound was identified based on the Glide score and protein-ligand interactions.

#### 3.1.3. Molecular alignment

In this study, the bioactive conformations of the ligands and their initial alignment were conducted *via* molecular docking. The results show that all derivatives are accurately positioned within the binding pocket, which facilitated the development of 3D-QSAR models. These docked conformations were subsequently exported and imported into SYBYL-X, where they underwent a systematic re-alignment to ensure that their relative spatial coordinates remained consistent throughout the modeling process.

#### 3.1.4. Construction of CoMFA and CoMSIA models

To construct the 3D-QSAR models, the 44 benzoxaborole derivatives were partitioned into a training set of 34 compounds and a test set of 10 compounds, maintaining an approximate ratio of 3.4 : 1. Selection was strategically performed to ensure a uniform distribution of biological activity and broad structural diversity, thereby enhancing the model's external applicability. The CoMFA and CoMSIA descriptors were calculated by placing the aligned molecular database within a 3D cubic lattice with a 2 Å grid spacing. For the CoMFA model, steric and electrostatic field energies were computed at each lattice intersection using Lennard-Jones and Coulomb potentials, respectively, with a sp^3^ hybridized carbon atom serving as the structural probe. Conversely, the molecular field energy function in the CoMSIA model takes the form of a distance-dependent Gaussian function. The contributions of the hydrogen bond acceptor, hydrogen bond donor, and hydrophobic fields were derived from the probe atom. The linear correlation between the CoMFA and CoMSIA domains and biological activity was analyzed using the partial least squares method.^42^ Cross-validation was performed with the leave-one-out method^43^ resulting in the cross-validation correlation coefficient (*q*^2^) and the optimal number of components (*N*). Additionally, the statistical significance of the models was assessed using the probability value (*F*), the standard error of estimate (SEE) and coefficient of determination (*r*^2^).

#### 3.1.5. Validation of the 3D-QSAR models

The predictive ability of the 3D-QSAR models was assessed using an external test set of 10 compounds. These test set compounds were also optimized and aligned as described previously, and their activity were predicted with the developed models. The modified *r*^2^ term (*r*_m_^2^), the external validation determination coefficient (*r*_pred_^2^) and the external standard deviation error of prediction (SDEP_ext_) were calculated to evaluate the performance of the models.

### 3.2. Molecular dynamics simulations

#### 3.2.1. Preparation of models

The initial docking hits were further evaluated through all-atom molecular dynamics (MD) simulations using the AMBER 24 (ref. 44) software package and subsequent binding free energy estimation with the ff19SB force field.^45^ Initially, the AM1 method^46^ was used to fully minimize the ligands. The electrostatic potentials were calculated at the HF/6-31G* level in Gaussian 09. The partial charges were determined by the RSEP fitting technique in AMBER. The general AMBER force field (GAFF2)^47^ through the Antechamber program was used to create the force-field parameters for the ligands. The LEaP module was used to assign hydrogen atoms, *via* which the ionizable residues were set to their default protonation states at neutral pH. The TIP3P water model^48^ was used to place each complex in a cubic box, maintaining a 10 Å minimum solute-wall distance, and counterions (Na^+^ and Cl^−^ at 0.15 M) were added to neutralize each system. Energy minimization of the solvated system was performed with 50 000 steps of steepest descent and 50 000 steps of conjugate gradient, using a nonbonded cutoff of 10 Å.

#### 3.2.2. Simulation protocol

The protocol for MD simulations consists of gradual heating, canonical ensemble (NVT) equilibration, isothermal-isobaric ensemble (NPT) equilibration, and a production process under NPT conditions (*T* = 310.15 K and *P* = 1 atm). More specifically, the system was heated gradually from 0 to 310.15 K over 50 ps, followed by NVT equilibrated at 310.15 K for 500 ps and further constant equilibration at 310.15 K for 500 ps. Then a process of equilibration procedure was conducted for each protein–ligand complex system until the system achieved a continuous stable status, *i.e.* production stage. During the total 200 ns MD run, the time step was set at 2 fs, with snapshots taken every 1 ps to record the conformational trajectory. Nonbonded interactions used a 10 Å cutoff, and the SHAKE algorithm^49^ constrained all bonds involving hydrogen atoms to their equilibrium lengths.

#### 3.2.3. MD trajectory analysis

The MD trajectories was analyzed by the Cpptraj code from AmberTools24.^50^ The fluctuations of each system and individual residues throughout the entire 200 ns simulations were examined to compute the RMSDs and RMSFs of the backbone atoms (CA) of CPSF3 using a mass-weighted average. For each MD trajectory, the first snapshot served as the reference structure. The LCPO algorithm^51^ was used to calculate the SASA of the ligand-binding site in each system, which tracked over time. For hydrogen bonds (X–H⋯Y) definition, the criteria are: (1) the angle between X–H in the hydrogen bond donor and Y in the hydrogen bond acceptor is greater than 120°; (2) the distance between atom X and atom Y is less than 3.5 Å.

#### 3.2.4. Binding free energy calculation

For each trajectory, the last 10 ns of simulations were extracted to calculate binding free energy. The molecular mechanics/Poisson–Boltzmann surface area (MM/PBSA) method^52^ was used to explore the energetic contributions to protein–ligand binding affinities. The binding free energy (Δ*G*_bind_) was calculated as follows:^53,54^Δ*G*_bind_ = *G*_complex_ − (*G*_protein_ + *G*_ligand_)where the energy term (Δ*G*_bind_) is estimated as follows:Δ*G*_bind_ = Δ*E*_gas_ + Δ*G*_sol_ − *T*Δ*S*Δ*E*_gas_ = Δ*E*_int_ + Δ*E*_ele_ + Δ*E*_vdw_Δ*G*_sol_ = Δ*G*_epb_ + Δ*G*_enpolar_In the above equations, the total binding free energy is decomposed into the gas-phase molecular mechanical energy (Δ*E*_gas_), the solvation free energy (Δ*G*_sol_), and the entropic contribution (−*T*Δ*S*). The gas-phase term (Δ*E*_gas_), is further partitioned into three distinct components: the internal energy (Δ*E*_int_), electrostatic interactions (Δ*E*_ele_), and van der Waals forces (Δ*E*_vdw_). The Δ*G*_sol_ was calculated using the continuous solvent method and consisted of a polar contribution (Δ*G*_epb_) and a non-polar contribution (Δ*G*_enpolar_). The change in conformational entropy, −*T*Δ*S*, was calculated by interaction entropy^55^ on a set of conformational snapshots taken from the MD trajectory. Additionally, to identify the key residues involved in the ligand binding process, the total free energy was decomposed for each residue in CPSF3.

## 4 Conclusions

In the present study, a combined strategy of docking-based 3D-QSAR and MD simulations was employed to explore the SARs of benzoxaborole analogs. The constructed CoMFA (*q*^2^ = 0.937, *r*^2^ = 0.826) and CoMSIA (*q*^2^ = 0.964, *r*^2^ = 0.882) models have yielded satisfactory statistical results. The acquired contour maps illuminated the SARs of 7-propanamide benzoxaboroles and effectively revealed critical structural determinants for CPSF3 binding and inhibition. The results of MD simulations revealed the critical molecular interactions between the inhibitors and CPSF3,such as metal-mediated coordination, Phe238-mediated π–π stacking, and van der Waals interactions and hydrophobic embedding mediated by hydrophobic amino acids. Guided by the obtained SAR, we designed a series of seven novel derivatives featuring the 7-propanamide benzoxaborole scaffold. Among them, compounds 50 and 51 show enhanced pIC_50_ values compared to compound 32, demonstrating that the QSAR models can actually be used.

This study, based on a human-vision-guided analysis of compound structures and their anti-proliferative activity against multiple cancer cell lines, provides a theoretical explanation for how 7-propanamide benzoxaboroles exerts its anti-MDA-MB-231 effect by inhibiting CPSF3, thereby revealing a conserved anti-cancer mechanism for this class of compounds. It is worth noting that, although these compounds show similar SAR trends across different cancer cell lines, they are not entirely consistent, reflecting the heterogeneity and complexity of different cancer cell lines. Therefore, further single-cell omics studies to compare the differences among these various cancer cell lines hold significant research value for the ongoing investigation and understanding of the mechanisms of action of these compounds, as well as for the discovery of other potential new targets.

## Author contributions

Liyang Ji: conceptualization, methodology, investigation, funding acquisition, resources, data curation, software, validation, formal analysis, writing – original draft, writing – review & editing, project administration, visualization. Yiwei Liu: methodology, investigation, funding acquisition, resources, data curation, software, validation, formal analysis, writing – review & editing, visualization. Guofeng Xu: resources, supervision, writing – review & editing, project administration.

## Conflicts of interest

The authors declare no conflict of interest.

## Supplementary Material

RA-OLF-D5RA10104B-s001

## Data Availability

The datasets generated and/or analyzed during the current study are available from the corresponding author on reasonable request. Supplementary information (SI) is available. See DOI: https://doi.org/10.1039/d5ra10104b.
