# Supplementary material for: Molecular modeling of 7-propanamide benzoxaboroles as CPSF3 inhibitors through docking-based 3D-QSAR and molecular dynamics simulations
Source: RSC Adv. 2026 Jul 2;16(34):32684–98. doi: 10.1039/d5ra10104b (PMC13325901; doi:10.1039/d5ra10104b)
Supplement: RA-016-D5RA10104B-s001 [file RA-016-D5RA10104B-s001.pdf]

Supplementary Information (SI) for RSC Advances.
This journal is © The Royal Society of Chemistry 2026

Molecular Modeling of 7-Propanamide Benzoxaboroles as CPSF3 Inhibitors through Docking-Based 3D-QSAR and Molecular Dynamics Simulations

Liyang Ji[†, *, a], Yiwei Liu[†, b] and Guofeng Xu[*, c]

[1] School of Pharmaceutical Sciences, Shanghai Jiao Tong University, Shanghai 200240, China; lyji2020@sjtu.edu.cn
[2] School of Chemical and Environmental Engineering and Shanghai Engineering Research Center of Green Fluoropharmaceutical Technology, Shanghai Institute of Technology, Shanghai 201418, China; yiweiliu@sit.edu.cn
[3] State Key Laboratory of Natural and Biomimetic Drugs, School of Pharmaceutical Sciences, Peking University, Beijing 100083, China; xuguofeng951006@outlook.com
* Correspondence: lyji2020@sjtu.edu.cn; xuguofeng951006@outlook.com

Table S1. Experimental pIC_{50} values (Exp.), predicted pIC_{50} values (Pred.) and corresponding residuals (Res.) of 7-propanamide benzoxaboroles as determined by the 3D-QSAR (CoMFA and CoMSIA) model.

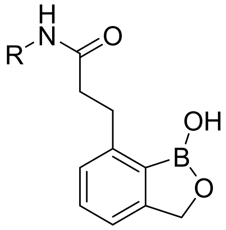

Comp.	R	pIC_{50}	CoMFA		CoMSIA	
		Exp	Pred	Res	Pred	Res
		**Training Set**				
**3**	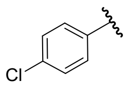	5.44	5.53	0.09	5.56	0.12
**4**	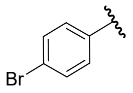	5.27	5.67	0.40	5.30	0.03
**6**	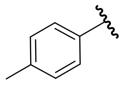	5.96	5.80	-0.16	5.93	-0.03
**7**	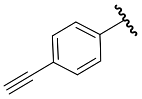	5.18	5.29	0.11	5.16	-0.02
**8**	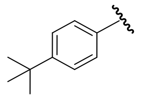	4.48	4.13	-0.35	4.49	0.01

**9**	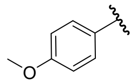	6.07	5.84	-0.23	6.07	0.00
**10**	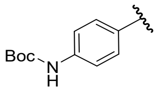	4.54	4.66	0.12	4.47	-0.07
**11**	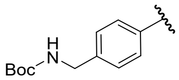	5.89	6.09	0.20	5.79	-0.09
**12**	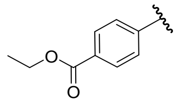	4.00	4.03	0.03	4.07	0.07
**14**	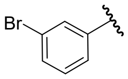	6.14	5.90	-0.24	5.99	-0.15
**16**	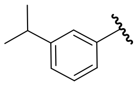	5.79	5.83	0.04	5.82	0.03
**17**	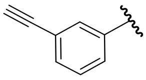	5.80	6.01	0.21	5.94	0.14
**18**	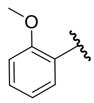	6.03	5.93	-0.10	6.01	-0.02
**19**	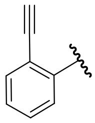	5.89	6.09	0.20	6.01	-0.05
**20**	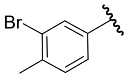	6.06	5.80	-0.26	6.01	-0.05
**21**	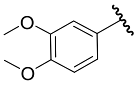	4.00	4.07	0.07	3.99	-0.01
**24**	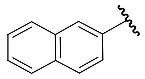	5.74	5.66	-0.08	5.72	-0.02
**26**	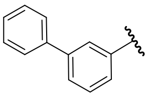	6.66	6.64	-0.02	6.70	0.04
**27**	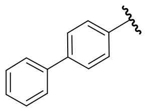	5.06	5.07	0.01	5.09	0.02
**30**	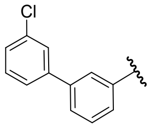	6.60	6.50	-0.10	6.49	-0.11
**31**	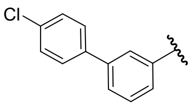	6.59	6.69	0.10	6.48	-0.11

**32**	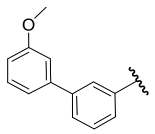	7.43	7.30	-0.13	7.35	-0.08
**33**	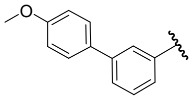	7.06	6.93	-0.13	6.92	-0.14
**34**	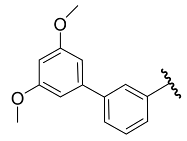	7.40	7.32	-0.08	7.48	0.08
**35**	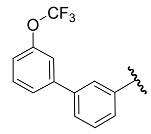	6.34	6.72	0.38	6.53	0.19
**36**	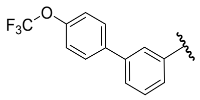	6.19	6.61	0.41	6.38	0.19
**37**	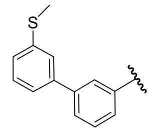	6.82	7.08	0.26	6.89	0.07
**38**	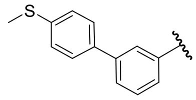	6.85	6.49	-0.36	6.87	0.02
**39**	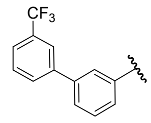	6.39	6.48	0.09	6.23	-0.16
**40**	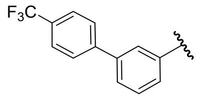	6.29	6.56	0.27	6.20	-0.09
**41**	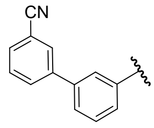	6.96	6.87	-0.09	6.93	-0.03
**42**	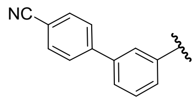	6.42	6.57	0.15	6.41	-0.01
**43**	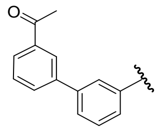	7.28	6.91	-0.37	7.41	0.13

**44**	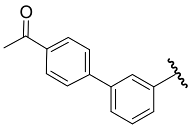	7.25	6.78	-0.47	7.20	-0.05
			**Test Set**			
**1**	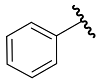	6.64	5.84	-0.80	5.98	-0.66
**2**	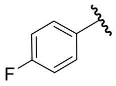	6.36	5.73	-0.62	5.67	-0.49
**5**	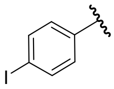	4.48	4.62	0.14	4.34	-0.14
**13**	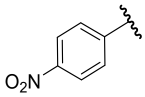	4.44	4.29	-0.05	4.38	-0.07
**15**	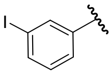	4.44	4.29	-0.05	4.37	-0.07
**22**	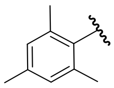	4.15	4.11	-0.04	3.86	-0.29
**23**	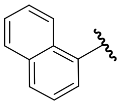	6.82	5.86	-0.96	6.55	-0.28
**25**	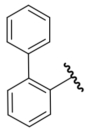	4.74	4.75	0.00	4.52	-0.22
**28**	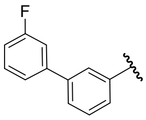	6.24	6.50	0.26	6.47	0.23
**29**	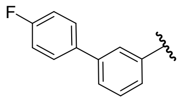	6.60	6.63	0.03	6.50	-0.10

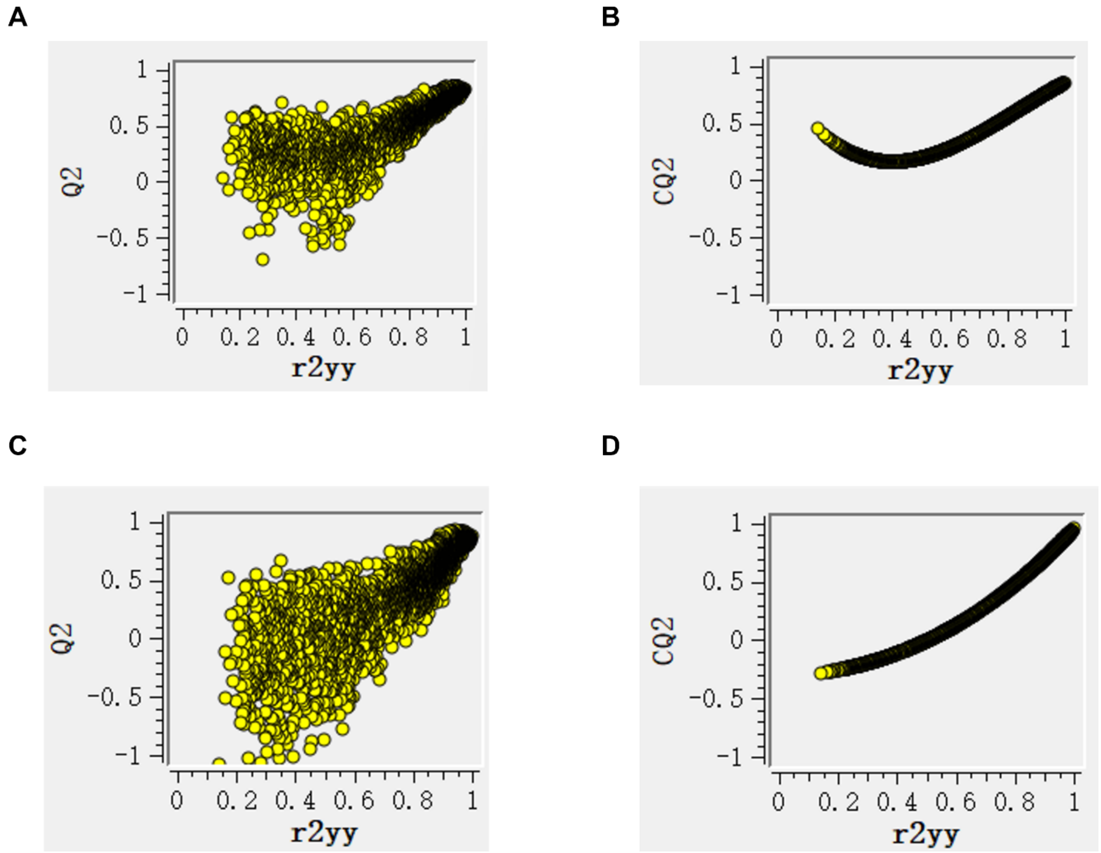

Fig. S1 Scrambling stability test analysis for CoMFA and CoMSIA models. The plots illustrate the dependence of Q^2 (or cQ^2) on the correlation coefficient (r^2yy) between the original and permuted biological activities. **(A)** Correlation plot of Q^2 versus r^2yy for the CoMFA model; **(B)** Linear regression of cQ^2 versus r^2yy for the CoMFA model; **(C)** Correlation plot of Q^2versus r^2yy for the CoMSIA model; **(D)** Linear regression of cQ^2 versus r^2yy for the CoMSIA model.

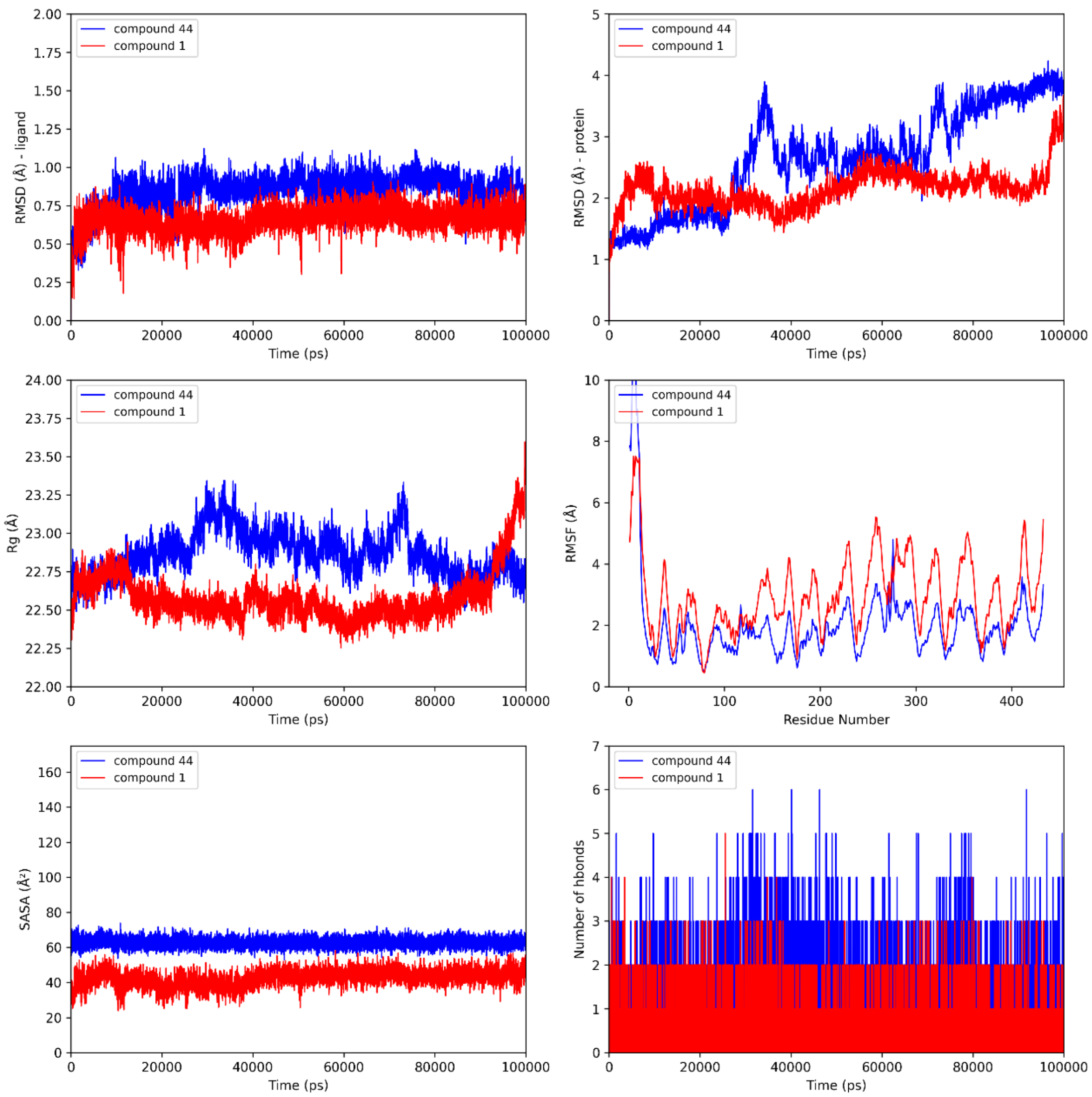

Fig. S2 RMSD-ligand (**A**), RMSD-protein (**B**), Rg (**C**), RMSF (**D**), SASA (**E**), and Number of H-bonds (**F**) for CPSF3-compound **1** and CPSF3-compound **44** complexes during the other simulation with a total duration of 100 ns. The CPSF3-compound **1** complex and CPSF3-compound **44** complex are shown in red and blue, respectively.

Table S2. Calculated binding energy (kcal/mol) for compounds **1** and **44** binding to CPSF3. Over the total simulation duration of 100 ns, a frame was sampled every 2 ns, resulting in a total of 50 frames.

Terms	CPSF3-compound 1	CPSF3-compound 44
ΔE_{vdw}	-21.348	-23.946
ΔE_{ele}	-352.147	-357.218
ΔG_{epb}	279.165	285.290
$\Delta G_{enpolar}$	-8.724	-8.602
ΔE_{gas}	-373.495	-381.161
ΔG_{sol}	288.495	286.688
$-T\Delta S$	/	/
ΔG_{bind}	-85.000	-94.473

$\Delta E_{gas} = \Delta E_{ele} + \Delta E_{vdw}$

$\Delta G_{sol} = \Delta G_{epb} + \Delta G_{enpolar}$

$\Delta G_{bind} = \Delta E_{gas} + \Delta G_{sol} - T\Delta S$
